# *anti*-1-(Benzyl­amino)­glyoxime. Corrigendum

**DOI:** 10.1107/S2056989021013530

**Published:** 2022-01-01

**Authors:** Yavuz Köysal, Şamil Işık, Nursabah Sarıkavaklı, Fatih Eyduran

**Affiliations:** a Department of Physics, Faculty of Arts and Sciences, Ondokuz Mayıs University, Kurupelit, 55139 Samsun, Turkey; b Department of Chemistry, Faculty of Arts and Sciences, Adnan Menderes University, Aydın, Turkey

## Abstract

In the paper by Köysal *et al.* [ *Acta Cryst.* (2004), E**60**, o515–o516], there was an error in the name of the last author.

## Abstract 2

Corrigendum to *Acta Cryst.* (2004), E**60**, o515–o516.

The name of the last author in the paper by Köysal *et al.* (2004 ▸) is incorrect and should be ‘Fatih Eyduran’, as given above.
